# Evaluation of workplace exposure to respirable crystalline silica in road construction industries in Alberta

**DOI:** 10.1177/07482337231176602

**Published:** 2023-05-31

**Authors:** Ariel Couture, Rose Marie Charuvil Elizabeth, Lianne Lefsrud, Fereshteh Sattari

**Affiliations:** 1 Department of Mechanical Engineering, 3158 University of Alberta, Edmonton, AB, Canada; 2 Department of Chemical and Materials Engineering, School of Engineering Safety and Risk Management, 3158 University of Alberta, Edmonton, AB, Canada

**Keywords:** Acute toxicity, occupational health, respiratory, air quality, lungs

## Abstract

Occupational exposure to respirable crystalline silica (RCS) is common for several occupations in construction, not only because of its presence in many handling materials but also in processes such as grinding and sawing. This study investigated workplace exposure to RCS as quartz in industries and occupations within road construction in Alberta through the RCS monitoring database provided by the Alberta Roadbuilders and Heavy Construction Association (ARHCA) between 2007 and 2016. Descriptive statistics were calculated for exposure-related variables, and mixed model analysis was performed to determine factors affecting the exposure levels. Results showed that the highest exposed workers were in the sand and gravel industry (GM = 45 μg/m^3^). For worker occupations, geometric means ranged from 78 μg/m^3^ for crusher operators to 10 μg/m^3^ for concrete truck operators. The maximum exposure severity was 33.3 times the occupational exposure limit (OEL) for the sand and gravel and 31 times the OEL for tower operators. The results also showed the effect of seasonal variability on RCS exposure levels. The heterogeneous exposure results indicated significant room for improvement and that controls should focus more on the activity performed than the occupation to lower exposure to RCS levels in industries.

## Introduction

Occupational exposure to respirable crystalline silica (RCS) in construction is one of the most at-risk industries, followed closely by mining and manufacturing (Radnoff et al., 2014; Bello et al., 2019). Overexposure to silica in construction has been reported in numerous studies, emphasizing the alarming levels of silica exposure among construction workers (Normohammadi et al., 2016; Doney et al., 2020; Yusoff et al., 2021). Many activities in construction are likely to expose workers to RCS, from material processing and handling to sandblasting and road milling, since silica is one of the main components of several construction materials such as cement, sand, and asphalt (Beaudry et al., 2013; Hammond et al., 2016). Despite recognizing occupations with high silica exposure, new exposure circumstances continue to be identified (Steenland and Ward, 2014). In addition, despite the development of the latest recognized and practical solutions, such as using effective local exhaust ventilation (LEV) (Mofidi et al., 2020), wet-cutting, and isolating dust (Park et al., 2019) to reduce RCS exposure in the construction industry, evaluation and control of exposure to RCS remains challenging due to variabilities in exposure determinants such as multiplicity of tasks within many trades, materials used, workforce mobility, and worksite characteristics (Beaudry et al., 2013; Sauvé et al., 2013; Li et al., 2019; Davies and Gorman-Ng, 2020; Khamraev et al., 2021).

Quartz is the most common type of naturally occurring crystalline silica, and because it is ubiquitous, there is a high potential for occupational exposure to RCS for workers in construction (Yeheyis et al., 2012). In a time of generally increasing concern about occupational diseases, several studies have quantified and found links between silica exposure and autoimmune disorders, renal diseases (Vupputuri et al., 2012), and rheumatoid arthritis (Mehri et al., 2020). To further emphasize the danger of exposure, RCS has been classified as a Group 1 carcinogen by the International Agency for Research on Cancer (IARC, 2022). According to CAREX (CARcinogen EXposure) Canada and Occupational Cancer Research Center (OCRC), 429,000 workers are potentially exposed to crystalline silica in Canada, contributing to around 2.4% of lung cancer cases annually (CAREX, 2016; OCRC, 2019). Approximately two million workers in the United States (Leung et al., 2012; Steenland and Ward, 2014), two million workers in Europe (Maciejewska, 2008), and more than 23 million workers in China (National Health Commission, 2018) are estimated to have been exposed to RCS. According to Chen et al. (2012), this caused 4.2% of deaths among industrial workers in China. However, the current exposure surveillance data for Alberta is not readily available. Therefore, estimating the exact number of workers at risk of exposure and the level of exposure to RCS in Alberta is challenging (Radnoff et al., 2014).

Government agencies worldwide have employed protective regulatory occupational exposure limits (OELs) (permissible exposure limit, or PEL, as used in the United States) to limit worker exposure concentrations to silica (Canadian Centre for Occupational Health and Safety (CCOHS), 2022). In Canada, the OEL ranges from 25 μg/m^3^ to 100 μg/m^3^, depending on the province and the specific polymorph of silica (cristobalite, Tripoli, quartz) (CAREX, 2023). However, in 2009, Alberta adopted 25 μg/m^3^ as the 8-hr OEL for crystalline silica as quartz (Lappi et al., 2014). Following the adoption of OEL for crystalline silica, Alberta industries changed their workplace exposure plans. Exposure measurements from 2007 to 2016 of various road-related construction industries were collected by Alberta Roadbuilders and Heavy Construction Association (ARHCA); however, no analysis was performed. This study, therefore, presents data collected from ARHCA and identifies the industries and occupations within road construction with the highest potential exposure to RCS using mixed-effects models.

## Methods

### Study design

A total of 634 RCS measurements representing six industries within the road construction industry and their respective occupations between 2007 and 2016 were obtained from ARHCA (Table 1). The database contained information about the industry type, occupation, RCS exposure values, year, and month of sample collection. The six industries were cement, sand and gravel (S&G), asphalt and paving (A&P), mechanic shop, surfacing, and concrete (Tables 2 and 3). A total of 18 occupations were identified in the database, and an ARCHA representative reclassified occupations of similar nature according to their standardized position list for ease of grouping samples (Table 4). In addition, the database also had RCS exposure values of area samples such as lunchroom, yard area, screen deck area, and miscellaneous (e.g., laboratory trailer).

**Table 1.** Descriptive statistics of respirable crystalline silica (RCS) exposure by year.

Year	N	AM (95% CI)	GM (95% CI)	GSD	Exceedance fraction, %
2007	88	64 (38–90)	33 (27–41)	2.72	53.4
2008	35	48 (31–65)	30 (21–42)	2.74	51.4
2009	20	66 (35–98)	37 (22–63)	3.33	65.0
2010	66	42 (31–53)	24 (19–31)	1.89	48.5
2011	35	51 (34–68)	30 (21–43)	2.93	51.4
2012	40	33 (18–47)	16 (12–23)	2.93	35.0
2013	93	97 (67–127)	57 (46–70)	1.93	74.2
2014	93	39 (24–54)	22 (18–26)	2.27	29.0
2015	156	50 (34–67)	46 (40–54)	2.32	39.1
2016	8	31 (18–45)	27 (17–42)	1.89	62.5

**Table 2.** Descriptive statistics of respirable crystalline fraction (RCS) exposure by industry.

Industry	N	AM (95% CI)	GM (95% CI)	GSD	Exceedance fraction
Sand and gravel	409	75 (63–87)	38 (34–42)	3.05	27.6%
Concrete	91	19 (14–23)	11 (7–17)	2.95	14.3%
Cement	67	26 (18–34)	16 (11–20)	2.63	29.9%
Asphalt and paving	37	28 (17–39)	18 (13–24)	2.48	29.7%
Surfacing	20	21 (13–29)	17 (12–23)	2.01	25%
Mechanic	10	27 (8–45)	19 (15–23)	2.38	40%
Total	**634**	—	—	—	—

**Table 3.** Exposure severity^a^ by industry type.

Industry	N	Mean	Median	IQR	Min.	Max.
Cement	67	1.047	0.480	1.000	0.080	5.960
Sand and gravel	409	3.002	1.280	2.400	0.156	33.28
Asphalt and paving	37	1.121	0.524	1.280	0.200	6.400
Mechanic shop	10	1.068	0.560	0.920	0.196	3.520
Surfacing	20	0.850	0.600	0.780	0.320	2.160
Concrete	91	0.747	0.440	0.412	0.020	6.000

^a^Severities were calculated by dividing the TWA exposure by the OEL.

**Table 4.** Descriptive statistics of respirable crystalline silica (RCS) exposure by occupation.

Occupation	N	AM (95% CI)	GM (95% CI)	GSD	Exceedance fraction
Crusher operator (CO)	110	125 (97–153)	78 (65–94)	2.62	86.4
Loader operator (LO)	94	28 (18–38)	16 (13–19)	2.56	24.5
Tower operator (TO)	92	79 (53–104)	43 (35–53)	2.86	66.3
Truck operator (TRO)	44	20 (12–28)	10 (7–15)	3.68	15.9
Plant operator (PO)	35	20 (10–30)	13 (10–17)	2.31	14.3
QC lab workers (QCWs)	32	57 (24–90)	27 (10–17)	3.47	43.8
Laborer (L)	24	9 (12–31)	15 (10–21)	2.34	25.0
Supervisor (S)	18	25 (17–32)	21 (15–28)	1.88	33.3
Maintenance worker (MW)	16	37 (20–53)	26 (16–40)	2.48	18.7
Batch mixer operator (BMO)	15	16 (8–24)	12 (8–18)	2.28	20.0
Groundsperson (GP)	12	30 (8–52)	18 (10–32)	2.79	33.3
Mechanic (M)	8	28 (4–28)	19 (10–36)	2.53	37.5
Excavator operator (EO)	8	25 (6–44)	18 (10–33)	2.27	25.0
Sweeper operator (SWO)	6	38 (26–42)	17 (7–46)	3.35	33.3
Grader operator (GO)	5	14 (7–20)	13 (9–19)	1.50	0
B-operator (BO)	5	28 (15–42)	26 (18–39)	1.56	60.0
Scale operator (SO)	4	25 (15–36)	20 (9–43)	2.23	50.0
Casting and coring operator (CCO)	4	24 (18–50)	17 (7–41)	2.48	25.0
Total	**532**	—	—	—	—

Exposure monitoring was performed independently by each company that participated either through a third-party consultant or by using trained in-house representatives. Personal sampling devices were provided to workers to wear throughout their workday to measure their exposure level to silica while performing their daily tasks. Participation in exposure assessments was voluntary; therefore, sequential participation was not consistent, resulting in varying samples in each industry. Air sampling measurements were collected over an eight-hour work shift, and the crystalline silica form studied was quartz. However, the type of sampling device used during the exposure assessments was not reported in the database. As directed by ARCHA, all samplings were performed according to National Institute for Occupation Safety and Health (NIOSH) method 7500, a standard method to measure workplace exposure to RCS.

Descriptive statistical analyses were used to calculate the arithmetic means (AMs) and geometric means (GMs) of RCS exposure levels in addition to 95% confidence intervals (CIs), geometric standard deviations (GSDs), and exceedance fractions of the six industries, 18 occupations, and four area samples in units of μg/m^3^. Exposure severities were calculated by dividing the time-weighted average (TWA) exposure by the OEL to facilitate the comparison of RCS exposures for each industry, occupation, and area concerning the Alberta OEL (Esswein et al., 2013; Radnoff et al., 2014).

### Mixed-effects models

Mixed-effects models have been widely accepted and applied in various scientific disciplines, such as economics (Blasco-Fontecilla et al., 2012), psychology (Magezi, 2015), and epidemiological studies (Kerckhoffs et al., 2022). The models are primarily used to describe relationships between a response variable and some covariates in data that are grouped according to one or more classification factors (Pinheiro and Bates, 2000). A major advantage of using mixed-effects models is that it allows examining the condition of interest while also considering variability between- and within-subjects (Oberg and Mahoney, 2007; Brown, 2021).

A mixed-effects model with random industry and occupation-specific (b) and measurement year as the fixed effect term (β) was adopted in this study to evaluate the association between exposure variables and RCS values. The model structure is described as follows:

(1) $$\mathrm{Ln}\left(Y\right)=\beta_{0}+\beta_{t}M++b_{i1-6}I+b_{o1-18}O+ɛ$$

where *β*_*0*_ is the model intercept, β_t_*T* is the continuous variable for the year of measurement, *b*_*i1-6*_*I* is the random effect term for industry type (b_1-6_), *b*_*o1-18*_*O* is the random effect term for occupation type (b_1-18_), and *ɛ* is the residual error. *I*, *O*, and *ɛ* were assumed statistically independent and normally distributed with mean 0 and variances *σ*_*bI*_^*2*^, *σ*_*bO*_^*2*^, and *σ*_*residual*_^*2*^, representing the between-industry, between-occupation, and residual variance components, respectively. The statistical model was developed using the “Proc Mixed” restricted maximum likelihood method and ‘Proc GLM’ procedure in Statistical Analysis Software (SAS) version 9.3 (SAS Institute Inc., Cary, N.C).

## Results and discussion

### Descriptive statistics

**Figure 1.**
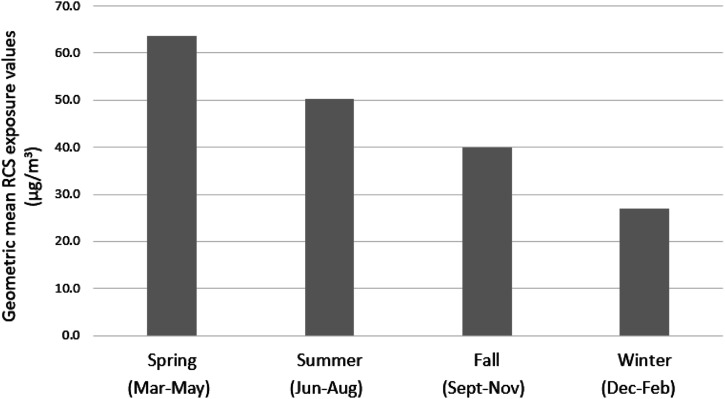
Mean respirable crystalline silica (RCS) exposure values by season.

Exposure analysis of the database showed that the highest level of exposure was recorded in 2013 (N = 93, GM = 57 μg/m^3^, 95% CI = 46–70 μg/m^3^), and the greatest number of measurements in 2015 (N = 156, GM = 46 μg/m^3^, 95% CI = 40–54 μg/m^3^) (Table 1). The highest GSD of 3.33 and exceedance fraction of 74.2% were recorded in 2009 and 2013, respectively. It is interesting to note that the highest RCS concentrations were measured during spring (63.7 μg/m^3^). This was followed by summer (50.3 μg/m^3^), fall (40.0 μg/m^3^), and winter (27 μg/m^3^) (Figure 1). These findings suggest that RCS concentrations are higher in warmer months, particularly in summer and spring, and lower during winters when the weather is cold.

The weather in the spring and summer seasons is generally dry, and RCS levels can be high in dry weather (Beaucham et al., 2018). This may have caused high exposure levels among construction workers. On the other hand, high relative humidity in the fall and winter seasons may have suppressed the dust levels because humid air decreases the time that dust is airborne and wet materials aerosolize at a lower rate causing low RCS exposure levels among workers (Sayler et al., 2018).

Several studies have found the effect of weather on silica exposure levels. For instance, Gottesfeld et al. (2008) found that average exposures of respirable particulate mass levels in Indian stone crusher mills were below PEL during the monsoon season; however, the levels exceeded the PEL in the dry season (summer). In Italy and Spain, Padoan et al. (2017) found higher road dust emissions in summer compared to winter, attributing it likely to lower pavement moisture. Furthermore, in a limestone mine industry in India, Mankar et al. (2019) reported that respirable dust concentrations and free silica levels were lower in the post-monsoon season than in the pre-monsoon season. This could be either because of reduced facility activity in winter or reduced emissions when the soil piles have higher moisture contents (Shiraki and Holmén, 2002).

#### Exposure to RCS by industry

The S&G industry, which is the sector that is traditionally considered to expose workers to elevated levels of RCS, had the highest mean RCS concentration measured (GM = 38 μg/m^3^, GSD = 3.05), and the lowest level of exposure was in the concrete industry (GM = 11 μg/m^3^, GSD = 2.95). The mechanic industry showed the highest exceedance fraction (40%); however, the lower end of 95% CI was greater than the 8-hr Alberta OEL (25 μg/m^3^) in the S&G industry, indicating a very high potential for overexposure in the industry (Table 2).

**Figure 2.**
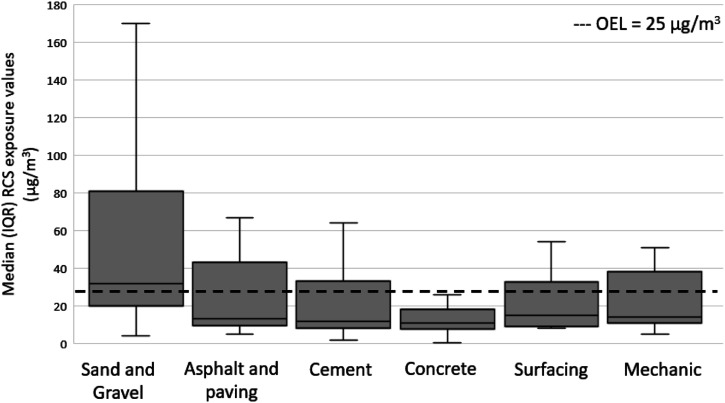
RCS exposure by industry compared with the Alberta OEL.S

The RCS exposure levels of industries within the road construction compared to Alberta’s 8-hr OEL are shown in Figure 2. It can be seen that no sample measurements from the concrete industry were above the OEL value. However, the median (30 μg/m^3^) exceeded the OEL (25 μg/m^3^) for the Sand and Gravel (S&G) industry. The positively skewed distribution indicates a few measurements had high RCS exposure values in all industries. Exposure values of RCS in the S&G industry (GM = 38 μg/m^3^) found in our study are similar to the concentrations reported by Sanderson et al. (2000) and Rando et al. (2001) in the US sand workers who found the values to be between 26 μg/m^3^ and 42 μg/m^3^. However, the concentrations in our study are about half the values reported in previous studies in the sand industry by Brown and Rushton (2005) in the United Kingdom and Radnoff et al. (2014) in Canada, who found the exposures value to be 90 μg/m^3^. The RCS values found in this study for the A&P industry (18 g/m^3^) were found to be higher than those reported by Hammond et al. (2016) in the United States, who found the RCS values to be between 4 μg/m^3^ and 9 μg/m^3^ among workers during asphalt pavement milling. The amount of crystalline silica exposed to workers is variable and can dependent on numerous factors such as the type of work performed, weather, duration and frequency of work activities, the material used for construction, and dust control measures (Carlo et al., 2010; Li et al., 2019). Variation in the work environments and conditions may have resulted in varying levels of silica exposure when compared to previous studies.

The S&G industry is quarrying and refining products with high silica content. Industrial silica sand is obtained from various sources, from a loose, unconsolidated granular state to hard, highly compacted rocks (Steenland and Sanderson, 2001). The different types of operations performed in the industry, such as crushing, screening, and milling, produce high amounts of silica dust in the air; therefore, the risk due to silica may be higher than in other quarrying industries (Brown and Rushton, 2005).

To compare and assess the magnitude of occupational exposure, exposure severities of industries were calculated (Table 3). The mean exceeded the median in every case, indicating that the severity data were positively skewed. The interquartile range (IQR) values show a large variability of RCS exposure, the highest seen in the S&G industry (2.400). The median severity measure was more significant than one in the S&G industry (1.280); however, all industry groups had minimum levels below one, indicating some samples below the OEL value. The maximum exposure severity was 33.28 times the OEL for the S&G industry due to very high exposures in the activities performed in the industry.

#### Exposure to RCS by occupation

The RCS exposure values for the 18 worker occupation groups encountered during the exposure assessment are shown in Table 4. Crusher operator had the highest mean RCS exposure levels (GM = 78 μg/m^3^) followed by tower operators (GM = 43 μg/m^3^) and quality control (QC) lab workers (GM = 27 μg/m^3^) likely reflecting an inadequate implementation of any preventive control measures to reduce workplace RCS concentrations (Table 4). For the crusher and tower operators, the lower end of 95% CI was greater than the 8-hr Alberta OEL (25 μm/m^3^), indicating a very high potential for overexposure in this occupation.

**Figure 3.**
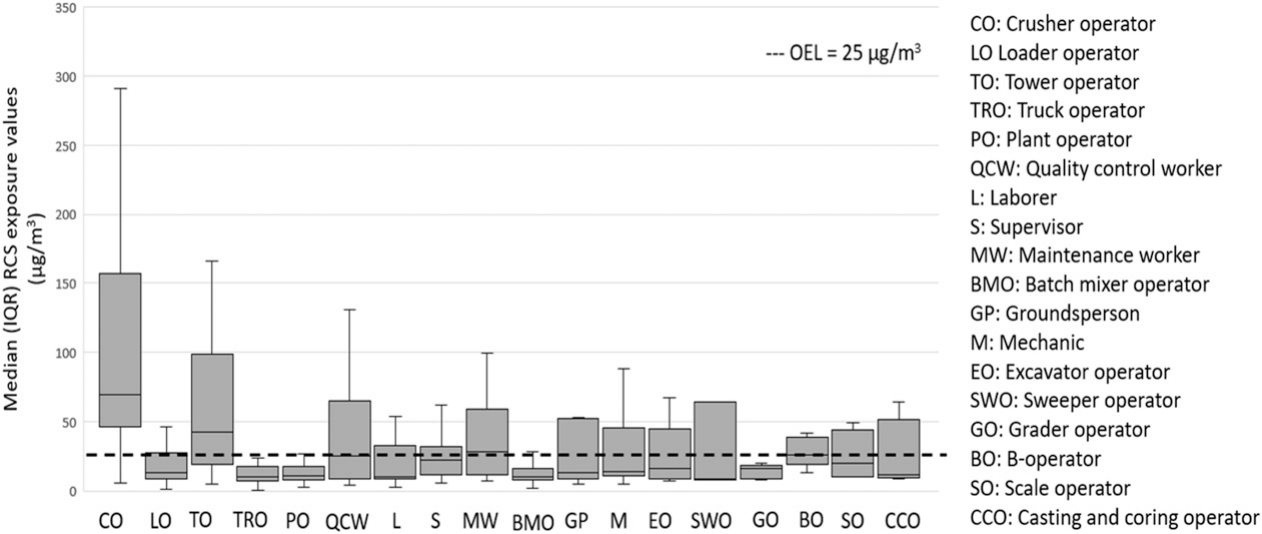
RCS exposure by occupation compared with the Alberta OEL.

The RCS exposure levels of occupations within the road construction industry are compared against Alberta 8-hr OEL and shown in Figure 3. For the crusher operator, the first quartile at 48 μg/m^3^ suggests that a significant proportion of the RCS measurements are above the established limit of 25 μg/m^3^. The results for RCS exposure values among crusher operators (78 μg/m^3^) in this study are lower than the study by Amran et al. (2017), who found that in crusher operators in Malaysian quarries, the RCS exposure values were 91 μg/m^3^. However, Mukhopadhyay et al. (2011) reported alarmingly high exposure levels to RCS among helper, loader, and feeder workers in the stone crushing facility in India, who found the values to be between 4510 μg/m^3^ and 8140 μg/m^3^, respectively. A study by Kuo et al. (2018) in Taiwan foundry workers in the sandblasting process identified the highest levels of both respirable dusts (520 μg/m^3^) and RCS (27 μg/m^3^). This may be explained because of differences in dust production from various processes that use different source materials, techniques, and dust control measures (Chen et al., 2012; Dahmann et al., 2008). Workers typically work with a variety of materials on different days and when worksites differ periodically, the exposure also differs. For instance, Nij et al. (2004) conducted a study on respirable dust and quartz exposure among construction workers and their findings revealed that the exposure model based on the type of material worked on, explained most of the between-worker variance when compared to the model based on job title. In addition, Grové et al. (2014) found statistically significant increase in respirable dust concentrations when coal miners performed a task in different positions in an underground production section.

Exposure severities by occupation were calculated, and the results are shown in Table 5. Similar to the results of severities by industry, the mean values of all occupations exceeded the median (except for grader operator), indicating that the severity data was positively skewed. The highest IQR values were observed in the crusher operator (4.440). The maximum exposure severity was observed in tower operators (31.6 times the OEL) and crusher operators (30.28 times the OEL).

**Table 5.** Exposure severity^a^ by occupation type.

Occupation	N	Mean	Median	IQR	Min.	Max.
Crusher operator (CO)	110	4.993	2.760	4.440	0.240	30.28
Loader operator (LO)	94	1.111	0.540	0.720	0.040	16.00
Tower operator (TO)	92	3.140	1.700	3.140	0.200	31.60
Truck operator (TRO)	44	0.800	0.420	0.416	0.020	5.280
Plant operator (PO)	35	0.808	0.440	0.408	0.096	6.000
QC lab workers (QCWs)	32	2.270	1.020	2.224	0.160	20.00
Laborer (L)	24	0.861	0.420	0.780	0.120	3.880
Supervisor (S)	18	0.982	0.880	0.800	0.240	2.480
Maintenance worker (MW)	16	2.660	1.120	2.400	0.2800	21.20
Batch mixer operator (BMO)	15	0.637	0.400	0.320	0.080	2.080
Groundsperson (GP)	12	1.199	0.532	1.720	0.200	4.800
Mechanic (M)	8	1.115	0.560	1.120	0.196	3.520
Excavator operator (EO)	8	1.005	0.660	1.080	0.276	2.680
Sweeper operator (SWO)	6	1.509	0.360	0.944	0.320	6.400
Grader operator (GO)	5	0.554	0.640	0.292	0.320	0.800
B-operator (BO)	5	1.128	1.040	0.400	0.520	1.680
Scale operator (SO)	4	0.990	0.800	1.180	0.400	1.960
Casting and coring operator (CCO)	4	0.970	0.480	1.180	0.360	2.560
Total	**532**	-	-	-	-	-

^a^Severities were calculated by dividing the TWA exposure by the OEL.

Equipment operators such as crusher and tower operators typically work in enclosed cabins, presenting a physical barrier to airborne particles, and yet our results showed high RCS exposure values. Employers should therefore ensure that even if operators are working in enclosed cabins, necessary control measures should be developed to eliminate or minimize occupational exposure to RCS. For instance, an engineering control in the hierarchy of controls in enclosed cabs with filtrations systems may lower the equipment operator’s exposure to RCS (Barlet et al., 2020). Rappaport et al. (2003) reported that using ventilated cabs reduced RCS exposures by ∼6-fold among operating engineers, and by using the wet dust suppression method, the RCS exposure values among laborers were reduced by 3-fold. Various levels of filtration can be incorporated into the heating, ventilation, and air conditioning (HVAC) system to maintain the ventilation quality of the air within the cab (Organiscak et al., 2013). In addition, QC lab workers generally spend most of their work time indoors, and their RCS exposure value (GM = 27 μg/m^3^) was found to be higher than the Alberta OEL. The exposures were likely due to the specific tasks the worker was doing at the time of assessment or incidental exposure from other activities at the work site. This suggests that when conducting an exposure assessment, careful attention should be given to the actual tasks performed by the workers and not just their occupation.

#### Exposure to RCS by area

Exposure to RCS in area samples showed that the lunchroom (GM = 31 μg/m^3^) and yard area (GM = 30 μg/m^3^) had the highest mean RCS exposure levels and exceedance fraction (Table 6). It is interesting to note such high exposure values in common places because workers usually do not have any protective equipment while they are indoors. Silica, when present in dust, can accumulate in their clothes, and when workers do not wear any protective equipment indoors, they become more vulnerable to risks.

**Table 6.** Descriptive statistics of RCS exposure by area.

Area	N	AM (95% CI)	GM (95% CI)	GSD	Exceedance fraction
Lunchroom	13	50 (20–82)	31 (8–45)	2.91	53.8%
Miscellaneous^a^	28	35 (21–48)	25 (13–48)	2.12	35.7%
Screen deck area	4	8 (15–30)	22 (18–27)	1.22	25.0%
Yard area	57	62 (31–93)	30 (23–40)	2.93	54.4%
Total	**102**	—	—	—	—

^a^Miscellaneous include samples from laboratory trailer, office rooms, etc.

Exposure severity analysis by area validated that lunchroom and yard areas have high RCS levels (Table 7). Cecala et al. (2013), in their study of the mining industry in the United States, found that within a 30-minute period, the respirable dust levels recorded in the lunchroom/break area were higher than outdoors. In addition, Haas and Cecala (2017) found five sources of respirable dust in the mining industry in the United States. The main source of indoor exposure was when workers got in and out of mobile equipment and used cloth chair seats that were located in offices or breakrooms. In addition, the authors found that the exposure levels were more evident when the cloth material was worn through and the foam seat padding was exposed. A second major source of respirable dust found by Haas and Cecala (2017) was to be in the dusty clothes and gloves of workers. When workers move on site while completing a task, their clothes may retain an excess amount of dust and may accumulate on indoor surfaces. In order to lower the exposure levels indoors, organizations should ensure good housekeeping. Workers should be encouraged to maintain general cleanliness inside the mobile cabs or when they are indoors. The organization should also replace any cloth chairs in the office or breakrooms present in their offices to reduce excess dust accumulation.

**Table 7.** Exposure severity^a^ by area.

Area	N	Mean	Median	IQR	Min.	Max.
Lunchroom	13	2.027	1.080	2.400	0.220	6.800
Miscellaneous^b^	28	1.386	0.800	0.480	0.240	6.400
Screen deck area	4	0.900	0.800	0.200	0.800	1.200
Yard area	57	2.464	1.200	0.760	0.156	31.600
Total	**102**	—	—	—	—	—

^a^Severities were calculated by dividing the TWA exposure by the OEL.

^b^Miscellaneous include samples from laboratory trailers, office rooms, etc.

### Mixed-effects model results

The parameter estimates derived from the mixed-effects models are shown in Table 8. An overall time trend of −4% per year was observed, and the random effects for industry showed that S&G and Cement were the industries with the highest exposure levels, while surfacing and A&P had the lowest exposure levels. Random effects for occupation showed that crusher operators and tower operators were the occupations with the highest exposure levels, while grader operators and laborers had the lowest exposure levels. Fixed effects in the model explained 19.8% of the between-industry variance and 45.4% of the between-occupation variance. In the final model, the residual variance was observed between industry (1.13) and between occupations (0.36); however, the largest value was due to unexplained variance (5.99), such as day-to-day variability.

**Table 8.** RCS exposure measurements used for statistical modeling, by model parameters.

*Fixed effect terms*		*β* ^a^	SE^b^	*p*-value
Intercept	—	−10.5	3.54	—
Year of measurement	Continuous years	−0.04	0.001	<0.0001
*Random effects terms*		BLUP^c^	SE^b^	*p*-value
Industry type	S&G	41.1	0.22	0.008
	A&P	−8.3	0.28	0.073
	Cement	−6.6	0.25	0.091
	Concrete	−7.5	0.24	0.093
	Surfacing	−11.7	0.33	0.126
	Mechanic	−7.1	0.39	0.084
Occupation type	Crusher operator	45.0	0.17	<0.001
	Loader operator	9.6	0.18	0.003
	Tower operator	17.1	0.17	<0.001
	Truck operator	−6.5	0.17	0.144
	Plant operator	−3.4	0.21	0.300
	QC lab workers	2.1	0.22	0.096
	Laborer	−10.1	0.24	0.076
	Supervisor	−2.7	0.26	0.105
	Maintenance worker	2.4	0.36	0.094
	Batch mixer operator	−6.5	0.28	0.134
	Groundsperson	−4.3	0.30	0.049
	Mechanic	−3.4	0.36	0.149
	Excavator operator	−6.6	0.36	0.037
	Sweeper operator	−9.8	0.41	0.106
	Grader operator	−10.1	0.34	0.057
	B-operator	0.68	0.36	0.035
	Scale operator	−10.2	0.26	0.029
	Casting and coring operator	−2.3	0.39	0.139
*Variance components*	Null model	Final model		%^d^
	VC^e^ SE^b^	VC^f^	SE^b^	
Between industry	1.41 0.025	1.13	0.031	19.8
Between occupation	0.66 0.43	0.36	0.143	45.4
Residual	6.22 0.03	5.99	0.03	3.69

^a^*β* for fixed effects derived from the mixed-effects model based on the restricted maximum likelihood method.

^b^SE = standard error.

^c^BLUP = best linear unbiased predictor for random effects.

^d^Percentage of variance explained by fixed effects.

^e^VC = variance components.

Overall, 49% of the exposed workers had an exposure value exceeding the Alberta OEL. These findings are similar to other studies in the United States and other countries (Hazrati et al., 2010; Hwang et al., 2017; Sanjel et al., 2018). The findings indicated a need for additional dust control interventions, such as engineering and administrative controls. Engineering controls protect workers either by removing hazardous working conditions or creating a barrier between the worker and the hazard. For RCS, common engineering controls used are wet methods, LEV, and physical barriers, which work to dampen or reduce the level of airborne particles that the worker could be exposed to as a result of a work process. Additional supporting data is provided by health and safety organizations, such as the Center for Disease Control and Prevention (CDC) and the NIOSH, which have an online database for Engineering and Physical Hazards Workplace Survey Reports (EPHWSR) collected across multiple industries, including construction (CDC, 2013). These workplace survey reports monitor and measure the effectiveness of task-specific engineering controls and are publicly available resources. While many of these surveys within the database involve analysis of LEV effectiveness during concrete grinding and cutting, a couple of reports measure the efficacy of the milling machine wet method and vacuum controls.

### Recommendations

• An engineering control that may effectively reduce RCS concentrations is to create a physical barrier between the worker and the hazard, such as machine guards, tarps, enclosed/covered conveyors, and curtains. Although engineering controls effectively mitigate exposure risk, they are often costly solutions upfront compared to the cost of administrative controls or personal protective equipment (PPE), as they may require replacement or alteration of the existing equipment. However, they are cost-effective over the long term.
• An administrative control could include educating or training workers about the hazards of silica, creating awareness, developing regular equipment maintenance schedules, and providing workers with the best management practices for managing and monitoring RCS exposure. Awareness of possible health risks and available preventive measures is essential for worker protection, as accurate risk perception leads to safer workplace behavior (Radnoff et al., 2015).
• Furthermore, employers could reduce occupational RCS exposure if a detailed risk assessment is performed for all activities on a worksite and exposure monitoring is performed for each employee potentially impacted. A strategic scheduling of tasks could be implemented to avoid scheduling work adjacent to silica-generating activities, as workers performing non-silica-generating tasks in the same work zone are equally at risk for overexposure as those performing the silica-generating task (Radnoff et al., 2015).
• Employers could also make employees aware of their potential risk of exposure and provide mitigative measures through exposure plans. By reviewing and providing training for these exposure plans, employers and employees would better understand the risks and impacts of exposure within their occupation and industry.
• Having a culture in which the employer regularly engages employees to participate in risk mitigation and prevention is critical in ensuring the protection of workers. If employees feel their perception of risk related to their health and safety at work is overlooked, they are more inclined to accept risks and reciprocate by exhibiting behaviors such as absenteeism and poor commitment (Amponsah-Tawiah and Mensah, 2016).
• Additionally, regular safety meetings involving workers’ participation can help generate new ideas for risk mitigation. By involving employees in developing and enforcing their safety processes, companies may see an increase in buy-in from employees who are more willing to accept changes made for their benefit. This involvement may lead workers to take the initiative for their safety or speak up when they see their colleagues not practicing safe work procedures or wearing their PPE.

## Study limitations

The primary limitation of this study is the lack of contextual information such as geographical location, the task performed, and the type of sampling device used for measurement. Contextual information usually contains a wealth of additional information, and this is beneficial because as more context is available, better recommendations can be provided. Consequently, the study could not analyze the influences of contextual factors on exposure levels of different occupations. Another limitation is the source of sample measurements. Data collection and reporting for ARCHA is mainly the employer’s responsibility, and it was observed that the number of observations differed by industry. Since participation in the exposure assessments was voluntary, silica exposure samples collected do not represent a random sample of exposure levels and the full range of industrial operations with potential exposures to RCS.

Despite the limitations, the findings provide a baseline for discerning patterns in occupational exposure to RCS in industries within road construction in Alberta and can be used as a guide for future prevention. Future data collection from industries should be more consistent with how samples are collected to assess the impacts of silica exposure better. This could be done by creating a well-structured exposure database containing contextual information. Standardizing testing methods and mandating monitoring requirements are additional ways to achieve consistent and reliable data. Standardization may include approving or providing specifications of sampling devices for the study, providing an approved list of laboratories in which the samples can be sent for analysis, and detailing what information is required with each sample. In addition, employers should ensure that they regularly measure and monitor the RCS concentrations to better control the exposure among workers. The current study found different RCS exposure levels in different seasons; however, further study is required to identify the impact of seasonal changes on occupational exposure to RCS.

## Conclusion

In conclusion, this study identified occupations within the road construction industry characterized by significant exposure to RCS in Alberta. Reducing RCS exposure levels under the Alberta OEL of 25 μg/m^3^ remains challenging. We described industries and occupations within road construction that are at high risk for RCS-related health problems. Some of the occupations, mainly equipment operators, should be the specific target of intervention programs to lower exposure to RCS. The analyses of RCS exposures in industries within the road construction industry provided substantial evidence that construction workers are overexposed relative to the OELs. The simple juxtaposition of measurements of RCS in industries is sufficient to document a pervasive problem with silica exposure and emphasize the need for improving existing control methods within the road construction industry in Alberta. Employers should regularly update their exposure control plans that meet the Occupational Health and Safety Regulation (OSHR) requirements. Workers’ risk perception and hazard awareness from workers can be further supplemented by actively creating a positive safety culture through regular policy and procedure reviews, frequent safety meetings, and engagement from all levels of the company hierarchy. Finally, high concentrations of RCS found in lunchrooms highlight the importance of documenting RCS exposure indoors and instituting adequate controls to reduce employee exposure. Any combination of these factors can be used by employers to protect workers from the risk of silica exposure that exists today. All tools in the occupational hygienists’ arsenal should be brought forward to bear on the problem, including any redesign of equipment, active methods of dust suppression, or short-term use of respiratory protection devices.
